# β_2_-Adrenergic Signaling Modulates Mitochondrial Function and Morphology in Skeletal Muscle in Response to Aerobic Exercise

**DOI:** 10.3390/cells10010146

**Published:** 2021-01-13

**Authors:** Vanessa Azevedo Voltarelli, Michael Coronado, Larissa Gonçalves Fernandes, Juliane Cruz Campos, Paulo Roberto Jannig, Julio Cesar Batista Ferreira, Giovanni Fajardo, Patricia Chakur Brum, Daniel Bernstein

**Affiliations:** 1 Department of Biodynamics of the Human Body Movement, School of Physical Education and Sport, University of São Paulo, São Paulo 05508-030, SP, Brazil; vanessa.voltarelli@alumni.usp.br (V.A.V.); larissafernandes@usp.br (L.G.F.); paulo.jannig@ki.se (P.R.J.); 2 Department of Pediatrics, School of Medicine, Stanford University, Palo Alto, CA 94304, USA; michael.j.coronado@gmail.com (M.C.); gfajardo@stanford.edu (G.F.); 3 Department of Anatomy, Institute of Biomedical Sciences, University of São Paulo, São Paulo 05508-030, SP, Brazil; jucampos@usp.br (J.C.C.); jcesarbf@usp.br (J.C.B.F.); 4 Department of Chemical and Systems Biology, School of Medicine, Stanford University, Palo Alto, CA 94304, USA

**Keywords:** aerobic exercise, mitochondria, β_2_-adrenoceptor, sympathetic nervous system, skeletal muscle

## Abstract

The molecular mechanisms underlying skeletal muscle mitochondrial adaptations induced by aerobic exercise (AE) are not fully understood. We have previously shown that AE induces mitochondrial adaptations in cardiac muscle, mediated by sympathetic stimulation. Since direct sympathetic innervation of neuromuscular junctions influences skeletal muscle homeostasis, we tested the hypothesis that β_2_-adrenergic receptor (β_2_-AR)-mediated sympathetic activation induces mitochondrial adaptations to AE in skeletal muscle. Male FVB mice were subjected to a single bout of AE on a treadmill (80% Vmax, 60 min) under β_2_-AR blockade with ICI 118,551 (ICI) or vehicle, and parameters of mitochondrial function and morphology/dynamics were evaluated. An acute bout of AE significantly increased maximal mitochondrial respiration in tibialis anterior (TA) isolated fiber bundles, which was prevented by β_2_-AR blockade. This increased mitochondrial function after AE was accompanied by a change in mitochondrial morphology towards fusion, associated with increased Mfn1 protein expression and activity. β_2_-AR blockade fully prevented the increase in Mfn1 activity and reduced mitochondrial elongation. To determine the mechanisms involved in mitochondrial modulation by β_2_-AR activation in skeletal muscle during AE, we used C2C12 myotubes, treated with the non-selective β-AR agonist isoproterenol (ISO) in the presence of the specific β_2_-AR antagonist ICI or during protein kinase A (PKA) and Gα_i_ protein blockade. Our in vitro data show that β-AR activation significantly increases mitochondrial respiration in myotubes, and this response was dependent on β_2_-AR activation through a Gα_s_-PKA signaling cascade. In conclusion, we provide evidence for AE-induced β_2_-AR activation as a major mechanism leading to alterations in mitochondria function and morphology/dynamics. β_2_-AR signaling is thus a key-signaling pathway that contributes to skeletal muscle plasticity in response to exercise.

## 1. Introduction

Skeletal muscle is one of the main tissues involved in acute and chronic adaptations induced by aerobic exercise (AE) and mitochondria are the major source of energy production [1]. It has been shown that AE induces mitochondrial biogenesis in skeletal muscle [2,3] and increasing evidence shows that AE can also modulate mitochondrial dynamics, inducing changes in mitochondrial morphology and function through processes of fusion and fission [4,5,6,7,8]. However, the cellular mechanisms underlying these AE-induced mitochondrial adaptations in skeletal muscle are still unclear.

In the last decade, many studies have shown that sympathetic responses and β-AR signaling can modulate expression of key proteins involved in mitochondrial biogenesis, fission, and fusion in skeletal muscle and other tissues [9,10,11]. Indeed, it has been recently reported that sympathetic neurons make close contact with neuromuscular junctions and form a network in skeletal muscle that functionally couple to different targets, including muscle fibers and organelles [12]. Since skeletal muscle adaptations to routine AE training are often driven by acute AE bouts, we hypothesize that exercise-induced sympathetic activation through β-adrenergic receptors (β-AR), mainly by the β_2_ subtype (~90% of total β-ARs in skeletal muscle) [13], may be one of the mechanisms modulating acute mitochondrial adaptations in skeletal muscle during AE [14,15]. We have previously shown marked mitochondrial remodeling in cardiac muscle in response to sympathetic stimulation during AE, a process which we have named “physiologic fragmentation” [16].

In the present study, we first tested in vivo the contribution of β_2_-ARs to skeletal muscle mitochondrial bioenergetics and dynamics during a single AE bout using pharmacologic inhibition. Next, using an in vitro approach, we evaluated which of the two main β_2_-AR signaling pathways: the canonical, mediated by Gα_s_ and protein kinase A (PKA); or the non-canonical, activated by Gα_i_, was responsible for these mitochondrial changes. The key findings of our study are that β_2_-AR activation enhances mitochondrial function in skeletal muscle during moderate to high-intensity AE through a Gα_s_-PKA-dependent pathway, while partially modulating mitochondrial morphology towards a fusion profile through increased Mitofusin1 (Mfn1) activity. In addition, our data show that the acute effects of sympathetic activation in skeletal muscle metabolism after an AE bout may be also due to a direct activation of β_2_-AR on skeletal muscle cells, and not only by the increased mobilization of energetic substrates from organs, tissues, and blood stream.

## 2. Materials and Methods

Animal model. Male FVB mice (3 months old) were housed in the animal facility of the School of Physical Education and Sport at University of Sao Paulo, with a temperature-controlled environment (22 °C) and in a 12:12-h dark-light cycle. Standard laboratory chow (Nuvital Nutrients, Curitiba, Brazil) and tap water were administered ad libitum. The sample size used for each experiment is indicated in the figures or figure legends. Mice were euthanized by cervical dislocation under isoflurane anesthesia, and tissues of interest were properly harvested and stored according to the analyses that they were submitted to. All procedures were performed in accordance with the Guide for the Care and Use of Laboratory Animals (National Institutes of Health, Bethesda, MD, USA) and with ethical principles in animal research adopted by the Brazilian Council for the Control of Animal Experimentation (CONCEA). In addition, this study was approved by the Ethical Committee of the School of Physical Education and Sport, University of Sao Paulo (protocol # 2015/06).

Running capacity test and aerobic exercise protocol. Aerobic exercise capacity was evaluated using a graded treadmill exercise protocol for mice as previously described [17]. Briefly, after being adapted to treadmill exercises over a week (10 min of exercise per session), mice were placed in the treadmill lane and allowed to acclimatize for at least 30 min. Intensity of exercise was increased by 3 m/min (6–45 m/min) every 3 min at 5° inclination until exhaustion. Exhaustion was defined as the moment when animals were unable to keep in pace with the treadmill for up to 1 min. AE bouts were performed at 80% of the maximal velocity (Vmax) achieved in the previous treadmill incremental maximal test, with 5° of inclination, for 60 min. The moderate-to-high intensity AE bout at 80% of Vmax was chosen in order to further stimulate catecholamines release during exercise, since it is known that hormones levels gradually increase according to AE intensity and duration [18,19]. All functional, structural, and biochemical analyzes were performed on tissues harvested right after (0 h) AE bouts.

Cell culture. C2C12 myoblasts (ATCC) were expanded in cell culture flasks (75 cm^2^, Corning Inc., Corning, NY, USA) containing DMEM (DMEM high glucose, Dulbecco’s modified Eagle’s Medium—Gibco, Invitrogen, Carlsbad, CA, USA), 10% fetal bovine serum (FBS, Gibco, Thermo Fisher Scientific, Waltham, MA, USA), antibiotics (penicillin, 100 U/mL–streptomycin, 100 µg/mL; Sigma-Aldrich, San Luis, MO, USA), and were incubated at 37 °C with 5% CO_2_. Differentiation protocol: when myoblasts confluence reached 90%, their differentiation into myotubes was initiated by switching the regular medium for a medium containing 2% of horse serum (HyClone Donor Equine Serum, GE Healthcare, Chicago, IL, USA), antibiotics (penicillin, 100 U/mL–streptomycin, 100 µg/mL; Sigma-Aldrich) and 1 µM of insulin (insulin from bovine pancreas, Sigma-Aldrich). After three days of differentiation, cells were treated for another three days with 10 µM of Ara-C (Cytosine β-D-arabinofuranoside, Sigma-Aldrich), an antitumor agent often used in C2C12 cell culture to remove remaining myoblasts after differentiation [20]. Ara-C was added to the differentiation medium, which was changed every day. On the sixth day of differentiation, serum was removed from the culture medium, and tests were performed on the following day. Trypsin-EDTA solution (Trypsin-EDTA solution 1x, Sigma-Aldrich) was used to remove the cells from the culture plate for 5 min, followed by centrifugation in culture medium for 5 min at 1000 rpm.

β-AR agonists treatment. Mice were treated with single doses of the selective β_2_-AR agonist formoterol (10 µg/kg, Sigma-Aldrich) [21], or the non-selective β-AR agonist isoproterenol (10 mg/kg, Sigma-Aldrich) [22,23,24], both for 30 min, administered via intraperitoneal injection. C2C12 myotubes were exposed to formoterol (10 µM) or isoproterenol (1 µM), both for 30 min.

Pharmacological blockade of β_2_-AR. Mice were treated with the selective β_2_-AR antagonist ICI 118,551 (10 mg/kg, Sigma-Aldrich) via intraperitoneal injection [25], 30 min before the AE bout, or 30 min before isoproterenol treatment. C2C12 myotubes were exposed to ICI 118,551 (300 nM) [26] for 60 min, considering that the last 30 min overlapped with the isoproterenol exposure.

Pharmacological blockade of PKA and Gα_i_ protein. C2C12 myotubes were exposed to the PKA inhibitor, PKI (5–24) (20 µM, Santa Cruz Biotechnology, Dallas, TX, USA) for 60 min [27], or to the Gα_i_ protein inhibitor, PTX (0.75 µg/mL—Pertussis toxin from *Bordetella pertussis*, Sigma-Aldrich) [27,28] for 4 h, considering that, in both treatments, the last 30 min overlapped with the isoproterenol exposure.

Mitochondrial function assessment. To evaluate mitochondrial function in mice skeletal muscle, the oxygen (O_2_) consumption test was performed in fiber bundles isolated from tibialis anterior (TA) muscle. The preparation of permeabilized fiber bundles and the O_2_ consumption test were performed as described by Anderson et al. (2009) [29]. TA muscle was harvested and immediately placed in iced isolation buffer, where fat and connective tissues were removed and, after that, small fiber bundles (~ wet weight: 1.5–2.0 mg) were gently separated along their longitudinal axis using fine tip tweezers. Subsequently, isolated fibers bundles were permeabilized with saponin (30 μg/mL), washed and maintained in assay buffer (4 °C) until the beginning of experiment (<2 h). To evaluate the O_2_ consumption rate (OCR) in fiber bundles, an Oroboros apparatus was used (OROBOROS O2K Oxygraph, Oroboros Instruments, Innsbruck, Austria), and the protocol was performed in assay buffer warmed at 37 °C. Basal OCR was measured in the presence of malate (2 mM), glutamate (10 mM), and succinate (10 mM), maximal OCR was determined after adding ADP (4 mM), and the ATP-independent OCR was evaluated after adding oligomycin (2 µg/mL). At the end of the assay, permeabilized fibers bundles were washed in ultrapure water to remove remaining salts, and then were weighed for subsequent data normalization. OCR is expressed as pmol O_2_/s/mg wet weight.

For the assessment of mitochondrial function in C2C12 cells, after the pharmacological treatments described above, myotubes were detached from culture flasks with trypsin-EDTA (Trypsin-EDTA solution 1x, Sigma-Aldrich), followed by centrifugation and resuspension in DMEM high glucose (Dulbecco’s-modified Eagle’s Medium—Gibco, Invitrogen), at 37 °C, free of serum, insulin, and antibiotics. The same medium was used as an assay buffer. After resuspension, cell counting was performed and 2 × 10^6^ cells (number determined by a previous titration performed under the same conditions) were added in Oroboros chamber (OROBOROS O2K Oxygraph, Oroboros Instruments) for running the assay. Basal OCR was determined only in the presence of the assay buffer, maximal OCR was determined after adding FCCP (0.25 µM), and the ATP-independent OCR was evaluated after adding oligomycin (4 µg/mL). OCR is expressed as pmol O_2_/s.

Isolation of skeletal muscle mitochondrial fraction. Enriched mitochondrial fraction was obtained from harvested hindlimb skeletal muscles, as previously described [30].

Immunoblotting. Protein expressions in total skeletal muscle lysate (RIPA buffer) and in mitochondrial fractions were evaluated by Western blotting. Briefly, samples were solubilized in sample buffer, separated on SDS-PAGE gel, and proteins were then transferred to a nitrocellulose membrane. After blocking nonspecific antigenic sites, membranes were incubated with primary and secondary antibodies, specific for each protein of interest. Primary antibodies: DRP1 (Becton Dickinson—BD, Franklin Lakes, NJ, USA), Fis1 (Enzo Life Sciences Inc., Farmingdale, NY, USA), Mfn1 (Abnova Corporation), Mfn2 (Abnova Corporation, Taipé, Taiwan), OPA1 (Becton Dickinson—BD), Enolase (Santa Cruz Biotechnology), and VDAC (Abcam, Cambridge, UK). Secondary antibodies: rabbit IgG and mouse IgG (GE Healthcare). Immunodetection was performed using the chemiluminescence method (Clarity™ Western ECL Blotting Substrate, Bio-Rad, Hercules, CA, USA). Quantitative blot analyses were performed using ImageJ (Scion Corporation, National Institute of Mental Health, NIH, Bethesda, MD, USA).

GTPase activity. Mfn1 was first immunoprecipitated from mice TA muscle lysate. GTPase activity was assessed using a GTPase Assay Kit (Innova Biosciences, Babraham Cambridge, Cambridgeshire, UK, 602). Briefly, the TA muscle lysate (250 mg protein) was incubated in 1 mL immunoprecipitation (IP) lysis buffer (150 mM NaCl, 5 mM EDTA, 10 mM Tris-HCl, 0.1% TritonX-100 (Sigma, X-100), pH 7.4) with antibody against Mfn1 for 3 h at 4 °C, followed by incubation with protein G PLUS-agarose beads (Santa Cruz Biotechnology, sc2002) for 1 h at 4 °C. After low speed centrifugation, 2300 g for 3 min at room temperature, the pellet was washed 3 times in 1 mL IP lysis buffer with low speed centrifugation after each wash. The immunoprecipitate was kept in 10 mL of IP lysis buffer. GTPase activity was normalized to the immunoprecipitate, measured by immunoblotting.

Mitochondrial morphology. Mitochondrial morphology was evaluated by transmission electron microscopy, and samples were prepared as previously described [31]. Mitochondrial number, area, perimeter, and elongation were quantified by ImageJ (Scion Corporation, NIH). Mitochondrial number was determined through the analysis of 5 image fields per animal (3 to 5 animals per group), and averages were presented as number of mitochondria per 100 μm^2^. Mitochondrial area, perimeter, and elongation were analyzed on 300 mitochondria per group.

ATP production in C2C12 myotubes. The ATP/ADP ratio was analyzed by bioluminescence using a commercial kit (ADP/ATP Ratio Assay Kit, Bioluminescent, Abcam), and assay was performed according to the manufacturer’s instructions.

Statistical analysis. According to each experimental design, non-paired Student’s *t* test or ANOVA one-way analysis, with Duncan post hoc, were used. *p* < 0.05 as significance level was considered.

## 3. Results

**Figure 1:**
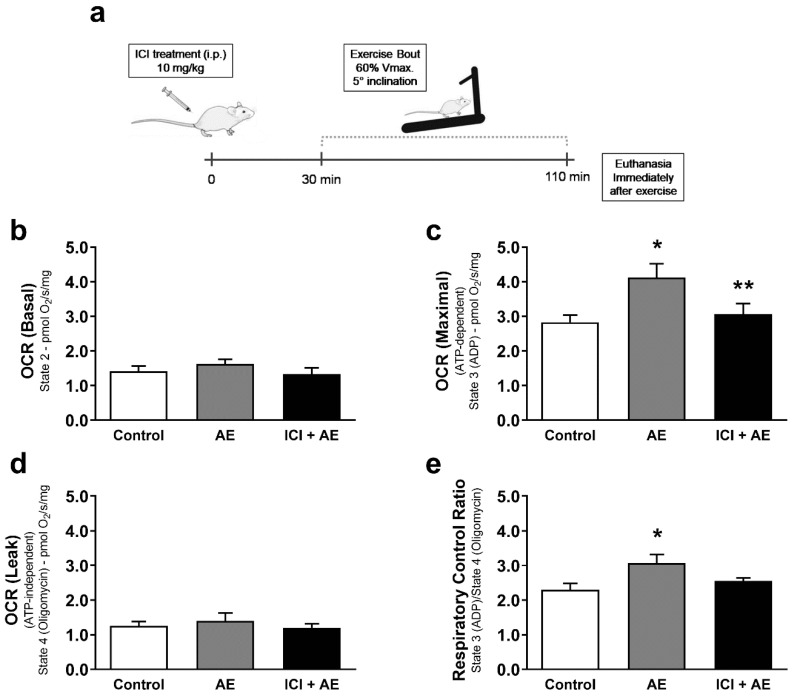
Exercise-induced maximal mitochondrial respiration is dependent on β_2_-adrenergic receptor (β_2_-AR) signaling. Experimental design for aerobic exercise under β_2_-AR blockade with ICI 118,551 (**a**), basal mitochondrial OCR (oxygen consumption ratio) (**b**), maximal mitochondrial OCR induced by ADP (**c**), ATP-independent OCR measured in the presence of oligomycin (**d**), and the respiratory control ratio (**e**) in permeabilized fiber bundles of tibialis anterior muscle isolated from exercised mice (single bout in a treadmill—80% of Vmax for 1 h, 5° inclination), and/or under β_2_-AR receptor blockade with ICI 118,551 (single dose—10 mg/kg, i.p., 30 min prior to exercise). Data are presented as mean ± SE. * *p* < 0.05 vs. control and ** *p* < 0.05 vs. AE. n = 5/group.

To test the role of muscle β_2_-AR signaling in vivo, we used a physiological approach of β_2_-AR activation during a single bout of AE. We hypothesized that exercise-induced β_2_-AR activation would lead to increased mitochondrial function and a change in mitochondria morphology. To test this hypothesis, we subjected mice to a single bout of AE in a treadmill (80% of Vmax for 60 min) under β_2_-AR blockade with the selective β_2_-AR antagonist, ICI 118,551 (ICI—10 mg/kg), or placebo (Figure 1a), and measured changes in mitochondrial function, morphology, and dynamics in tibialis anterior muscle (TA) (chosen because of its predominantly glycolytic metabolism).

Exercised mice showed no significant change in both basal and ATP-independent (under ATP synthase inhibition with oligomycin) mitochondrial respiration (Figure 1b), while developing enhanced maximal mitochondrial respiration after ADP addition (Figure 1c), and an increased respiratory control ratio (RCR) (Figure 1e) in fiber bundles isolated from TA. Performing AE under β_2_-AR blockade significantly decreased the maximal oxygen consumption rate (OCR), and prevented the increase in RCR observed in AE without blockade (Figure 1c,e). These data indicate that β_2_-AR signaling plays a key role in skeletal muscle mitochondrial function during AE.

**Figure 2:**
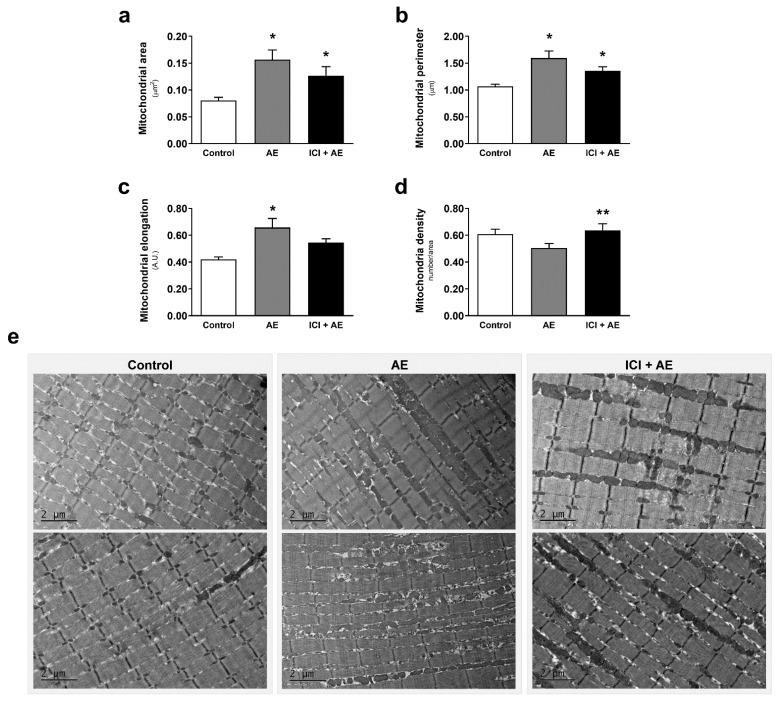
Skeletal muscle mitochondrial morphology is modulated by aerobic exercise, partially mediated by β_2_-ARs. Mitochondrial area (**a**), perimeter (**b**), elongation (**c**), mitochondria number per area (**d**), and electron microscopic representative images (**e**) of tibialis anterior muscle from exercised mice (single bout in a treadmill—80% of Vmax for 1 h, 5° inclination), with or without β_2_-AR blockade with ICI 118,551 (single dose—10 mg/kg, i.p., 30 min prior exercise). Data are presented as mean ± SE. * *p* < 0.05 vs. control and ** *p* < 0.05 vs. AE. n = 5/group.

The enhanced maximal mitochondrial respiration in the AE group was associated with an increase in mitochondrial fusion, represented by increased mitochondrial area, perimeter, and elongation in TA (Figure 2a–c). Exercise-induced mitochondrial fusion was partially prevented by blocking β_2_-AR signaling with ICI, which was manifested as less elongated mitochondria in ICI+AE animals (Figure 2c). Interestingly, this response was restricted to glycolytic muscles, since the same bout of AE did not induce significant changes in mitochondria morphology in soleus muscle (aerobic skeletal muscle with predominant type I oxidative fibers) (Figure S2), which depends predominantly on oxidative metabolism. It is worth mentioning that ICI treatment alone did not induce any mitochondrial morphology change in TA compared with control group (Figure S1).

**Figure 3:**
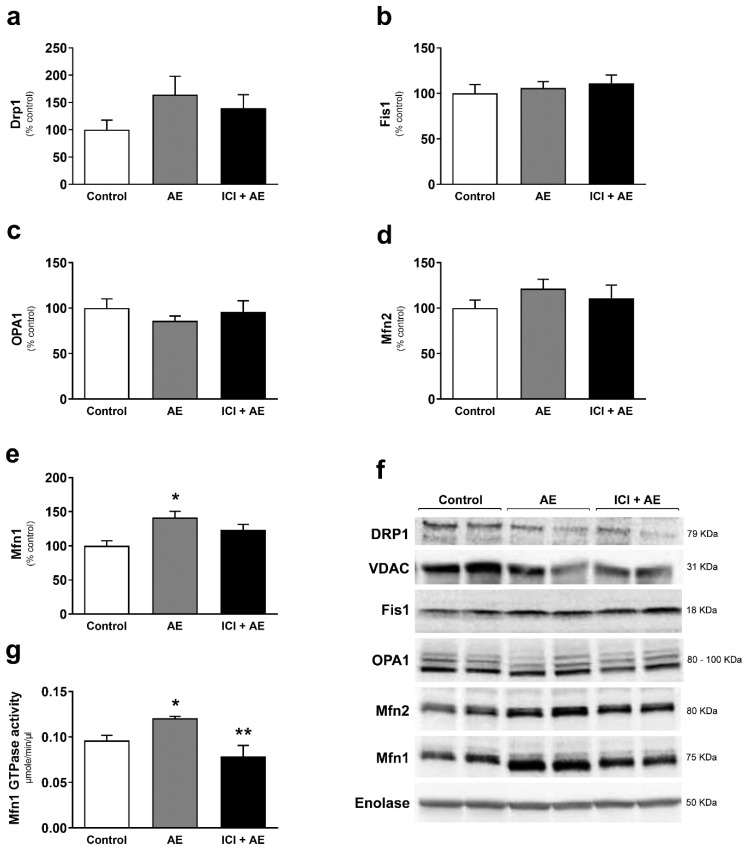
Exercise-induced skeletal muscle mitochondrial fusion is associated with increased Mfn1 expression and activity, mediated by β_2_-ARs. DRP1 (mitochondrial fraction) (**a**), Fis1 (**b**), OPA1 (**c**), Mfn2 (**d**), and Mfn1 (**e**) protein expression, immunoblotting representative images (**f**), and Mfn1GTPase activity (**g**) in tibialis anterior muscles from exercised mice (single bout in a treadmill—80% of Vmax for 1 h, 5° inclination), with or without β_2_-AR receptor blockade with ICI 118,551 (single dose—10 mg/kg, i.p., 30 min prior exercise). Enolase and VDAC were used as loading control for total lysate and mitochondrial fraction protein expression, respectively. Data are presented as mean ± SE. * *p* < 0.05 vs. control and ** *p* < 0.05 vs. AE. n = 5/group.

To confirm changes in mitochondrial dynamics, we next evaluated expression of dynamic regulators in TA and found that AE-induced mitochondrial fusion was associated with significantly increased Mfn1 protein expression (Figure 3e) and no significant change in Mfn2, OPA1, Fis1, or DRP1 (Figure 3a–d). Blocking β_2_-ARs with ICI reduced the increase in Mfn1 expression (Figure 3e–f). To further confirm the role of Mfn1 in AE-induced mitochondrial elongation, we immunoprecipitated Mfn1 (Figure S2) and measured Mfn1 GTPase activity, which was increased in TA after AE (Figure 3g). Performing exercise under β_2_-AR blockade prevented this increase. These data suggest that β_2_-AR signaling enhances Mfn1 activity during a bout of AE.

**Figure 4:**
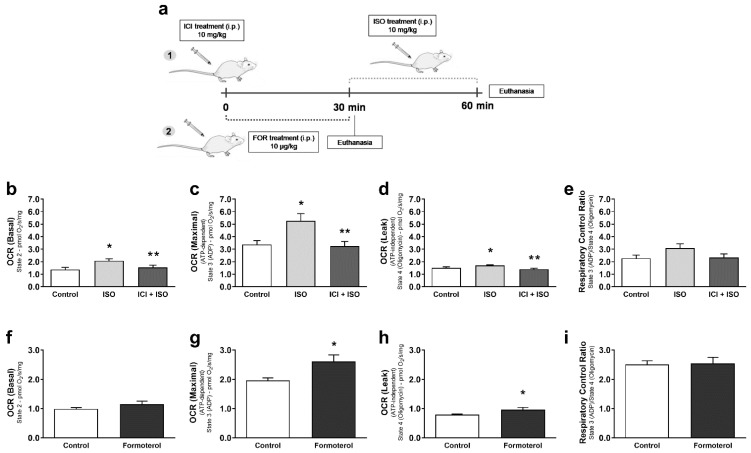
Pharmacological activation of β_2_-AR receptors leads to increased mitochondrial respiration in skeletal muscle. Experimental design for pharmacological treatments in mice (**a**), basal mitochondrial OCR (oxygen consumption ratio) (**b**), maximal mitochondrial OCR induced by ADP (**c**), ATP-independent OCR measured in the presence of oligomycin (**d**), and the respiratory control ratio (**e**) in permeabilized fiber bundles of tibialis anterior muscle isolated from mice treated with the non-selective β-AR agonist isoproterenol (ISO—single dose—10 mg/kg, i.p. 30 min), with or without β_2_-AR receptor blockade with ICI 118,551 (single dose—10 mg/kg, i.p., 30 min prior to ISO treatment). Basal mitochondrial OCR (**f**), maximal mitochondrial OCR induced by ADP (**g**), ATP-independent OCR measured in the presence of oligomycin (**h**), and the respiratory control ratio (**i**) in permeabilized fiber bundles of tibialis anterior muscle isolated from mice treated with the selective β_2_-AR agonist formoterol (FOR-single dose—10 µg/kg, i.p., 30 min). Data are presented as mean ± SE. * *p* < 0.05 vs. control and ** *p* < 0.05 vs. ISO. n = 5/group.

We next used a pharmacological approach to determine whether β_2_-AR signaling can modulate skeletal muscle mitochondrial function and dynamics in vivo in the absence of exercise. We stimulated β_2_-AR signaling with formoterol (10 μg/kg), a selective β_2_-AR agonist, or isoproterenol (ISO, 10 mg/kg), a non-selective β1-AR and β2-AR agonist for 30 min. Since ISO is a non-selective agonist, we re-confirmed the role of β_2_-AR signaling by pretreated ISO-exposed mice with ICI (10 mg/kg), for selective β_2_-AR antagonism. After formoterol and ISO treatments, TA fiber bundles were isolated for mitochondrial respiration assessment. Only ISO treatment induced an increase in basal OCR (Figure 4b,f), while both ISO and formoterol treatments significantly increased maximal (Figure 4c,g) and ATP-independent (Figure 4d,h) mitochondrial respiration. In contrast with AE data, no significant differences in RCR were shown between groups for ISO and formoterol treatments (Figure 4e,i). Blocking β_2_-ARs with ICI significantly reduced the increased basal, maximal, and ATP-independent mitochondrial respiration induced by ISO treatment (Figure 4b–d), indicating again that the β_2_-AR subtype is playing a major role in modulating mitochondrial function during pharmacologically simulate AE.

**Figure 5:**
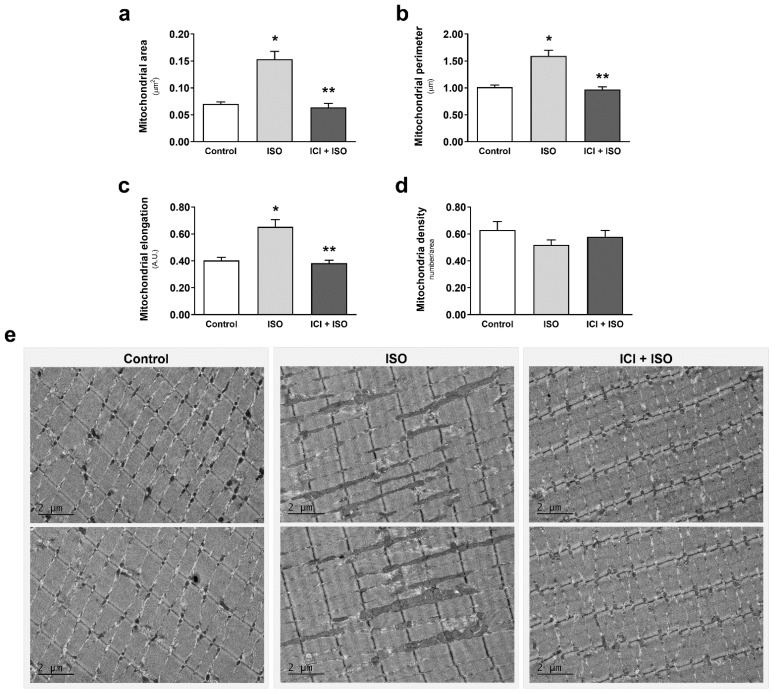
Pharmacological activation of β_2_-ARs leads to mitochondrial fusion in skeletal muscle. Mitochondrial area (**a**), perimeter (**b**), elongation (**c**), mitochondria number per area (**d**), and electron microscopy representative images (**e**) of tibialis anterior muscle from mice treated with the non-selective β-AR agonist isoproterenol (ISO—single dose—10 mg/kg, i.p. 30 min), with or without β_2_-AR receptor blockade with ICI 118,551 (single dose—10 mg/kg, i.p., 30 min prior to ISO treatment). Data are presented as mean ± SE. * *p* < 0.05 vs. control and ** *p* < 0.05 vs. ISO. n = 5/group.

**Figure 6:**
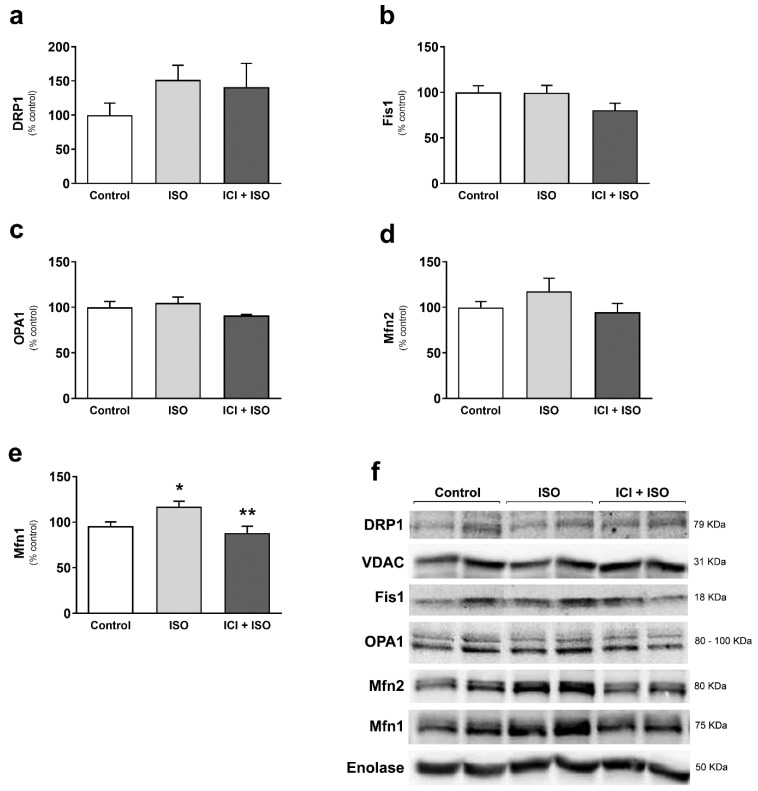
Pharmacological activation of β_2_-ARs increases Mfn1 expression in skeletal muscle. DRP1 (mitochondrial fraction) (**a**), Fis1 (**b**), OPA1 (**c**), Mfn2 (**d**), and Mfn1 (**e**) protein expression, and immunoblot representative images (**f**) in tibialis anterior muscles from mice treated with the non-selective β-AR agonist isoproterenol (ISO—single dose—10 mg/kg, i.p. 30 min), with or without β_2_-AR receptor blockade with ICI 118,551 (single dose—10 mg/kg, i.p., 30 min prior to ISO treatment). Enolase and VDAC were used as loading control for total lysate and mitochondrial fraction protein expression, respectively. Data are presented as mean ± SE. * *p* < 0.05 vs. control and ** *p* < 0.05 vs. ISO. n = 5/group.

TA muscles were also imaged by electron microscopy to assess changes in mitochondrial morphology. β-AR stimulation with ISO activated mitochondrial fusion, evidenced by a significant increase in mitochondrial area, perimeter, and elongation (Figure 5a–c), with no changes in mitochondrial number (Figure 5d). Co-treatment with ICI blocked ISO-induced mitochondrial fusion (Figure 5a–c), demonstrating the important role of β2-AR signaling in regulating mitochondrial morphology in TA. To confirm activation of β-AR-induced mitochondrial fusion, we measured changes in mitochondrial dynamics protein mediators. β-AR stimulation with ISO resulted in a significant increase in Mfn1 (Figure 6a), which was blocked with co-treatment with ICI (Figure 6e). There were no changes in Drp1, Fis1, OPA1, and Mfn2 protein expression (Figure 6a–d). These results corroborate our protein expression data from exercised mice and confirm that activation of β_2_-AR signaling in glycolytic muscle induces mitochondrial fusion and enhances mitochondrial function in vivo.

**Figure 7:**
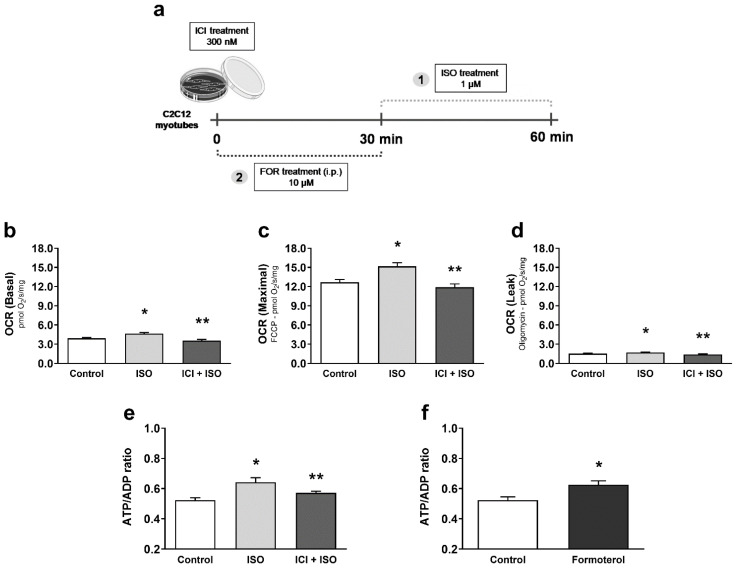
Pharmacological activation of β_2_-AR receptors leads to increased mitochondrial function and ATP production in C2C12 myotubes. Experimental design for pharmacological treatments in C2C12 myotubes (**a**), basal mitochondrial OCR (oxygen consumption ratio) (**b**), maximal mitochondrial OCR induced by FCCP (**c**), ATP-independent OCR measured in the presence of oligomycin (**d**), and ATP production (**e**) in C2C12 myotubes acutely exposed to the non-selective β-AR agonist isoproterenol (ISO—1 µM for 30 min), with or without β_2_-AR receptor blockade with ICI 118,551 (300 nM, 30 min prior to ISO treatment). ATP production (**f**) in C2C12 myotubes acutely exposed to the selective β_2_-AR agonist formoterol (10 µM for 30 min). Data are presented as mean ± SE. * *p* < 0.05 vs. control and ** *p* < 0.05 vs. ISO. n = 8/group.

To further confirm the mechanism for β2-AR-induced mitochondrial fusion and enhanced function in skeletal muscle, we utilized C2C12 mouse myoblast-derived myotubes and modulated β-AR signaling with the above agonists and antagonist. After myotube differentiation (Figure S4; MHC Type II protein expression in skeletal muscle, C2C12 myoblasts and C2C12 myotubes after 72 h of differentiation), cells were treated with ISO for 30 min (1 µM), in the presence or absence of ICI (300 nM) (Figure 7a) and mitochondrial function was measured by Oroboros Oxygraph. β-AR simulation with ISO significantly increased both basal, maximal, and ATP-independent mitochondrial respiration in C2C12 myotubes, which were blocked by co-treatment with ICI (Figure 7b–d). Since both ISO and formoterol treatments increased the ATP-independent OCR, we intended to determine if the increased mitochondrial function observed after pharmacological treatments would also be coupled to ATP production. To answer this question, we first measured ATP/ADP ratio after ISO treatment in myotubes and found an increased ATP/ADP ratio, which was also significantly blocked by ICI (Figure 7e). To further confirm the role of β2-AR signaling, we also treated C2C12 myotubes with formoterol (10 µM) for 30 min (Figure 7a) and observed a similar significant increase in the ATP/ADP ratio (Figure 7f). These data, mirroring our in vivo data, further support the role of β_2_-AR signaling in enhancing mitochondrial function in skeletal muscle cells. 

**Figure 8:**
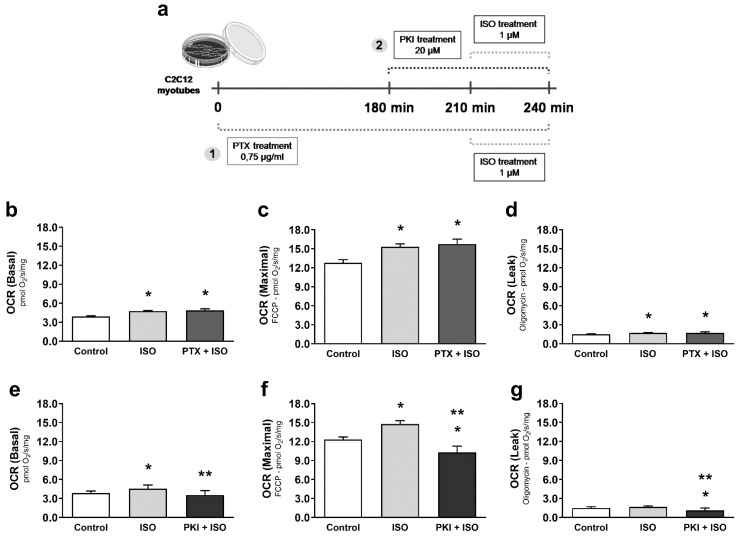
Increased mitochondrial function after activation of β_2_-ARs is dependent on downstream Gα_s_-PKA signaling. Experimental design for pharmacological treatments in C2C12 myotubes (**a**), basal mitochondrial OCR (oxygen consumption ratio) (**b**), maximal mitochondrial OCR induced by FCCP (**c**), and ATP-independent OCR measured in the presence of oligomycin (**d**) in C2C12 myotubes acutely exposed to the non-selective β-AR agonist isoproterenol (ISO—1 µM for 30 min), with or without Gα_i_ protein blockade with pertussis toxin (PTX—0.75 µg/mL, 3 h prior to ISO treatment). Basal mitochondrial OCR (**e**), maximal mitochondrial OCR induced by FCCP (**f**), and ATP-independent OCR measured in the presence of oligomycin (**g**) in C2C12 myotubes acutely exposed to the non-selective β-AR agonist isoproterenol (ISO—1 µM for 30 min), and/or under PKA blockade with PKI (20 µM, 30 min prior to ISO treatment). Data are presented as mean ± SE. * *p* < 0.05 vs. control and ** *p* < 0.05 vs. ISO. *n* = 9/group.

The β_2_-AR has several important downstream pathways that determine endpoint responses: β_2_-ARs coupled to Gα_s_ resulting in protein kinase A (PKA) activation and β_2_-ARs coupled to Gα_i_ resulting in β-arrestin activation. To identify which downstream pathway is responsible for modulating mitochondrial function during simulated AE, C2C12 myotubes were treated with ISO under the following conditions: blockade of Gα_s_ signaling by PKA inhibition with PKI; or Gα_i_ blockade with pertussis toxin (PTX). The ISO-induced increase in mitochondrial function was unaffected by Gα_i_ blockade with PTX (Figure 8b–d). In contrast, blockade of PKA with PKI significantly reduced mitochondrial function (Figure 8e–g). These results indicate that β_2_-AR activation enhances skeletal muscle mitochondrial function through Gα_s_ and PKA downstream signaling pathways.

## 4. Discussion

We report that β_2_-AR activation plays a key role in acute mitochondrial adaptations in skeletal muscle after a single bout of AE. Evidence supporting this finding includes: (i) performing AE under β_2_-AR blockade with ICI blunts the enhanced maximal mitochondrial function in skeletal muscle induced by AE, whereas pharmacological stimulation of β_2_-ARs also increases skeletal muscle mitochondrial function; (ii) a single bout of moderate-to-high intensity AE, or acute ISO treatment, changes mitochondrial morphology towards fusion in skeletal muscle through increased Mfn1 expression and activity. Blocking β_2_-ARs partially prevented these AE effects; and (iii) the improved mitochondrial function after pharmacological activation of β_2_-ARs in C2C12 myotubes is dependent on the Gα_s_-PKA signaling pathway. 

Our results corroborate several prior studies that demonstrated increased mitochondrial function induced by a single bout of AE in skeletal muscle. However, the effect of acute exercise on mitochondria respiration is still under investigation and the results have often been contradictory [32,33,34,35,36]. This might be related to the specific skeletal muscle studied, since muscles comprised of glycolytic fibers are more sensitive to sympathetic signaling and depend upon rapid neuromuscular junction transmission, which is innervated by sympathetic fibers [12,21,37,38,39,40].

The increased maximal mitochondrial respiration after AE was dependent on sympathetic activation through β_2_-ARs in TA. This is of interest, if one considers the increasing contribution of glycolytic muscles to provide energy for moderate-to-high intensity sustained exercise. In addition, some well-known mechanisms underlining the enhanced mitochondrial function during and after an AE bout, such as increased AMP/ATP ratio and an increased NADH and calcium influx to the mitochondrial matrix, are modulated by the sympathetic activation [1,41,42,43]. Considering the significant blockade of the increased maximal OCR with ICI during AE, this suggests that these mechanisms are dependent on the sympathetic activation in skeletal muscle. It is worth highlighting the clinical implications of this β_2_-AR mechanism underlying mitochondrial adaptation to chronic AE in skeletal muscle, as depending on the situation, one might consider this response before choosing β-blockers in clinical settings. In fact, we have previously demonstrated that lack of β_2_-ARs aggravates heart failure-induced skeletal muscle myopathy in mice [44]. 

In the present study, we further demonstrate that an acute pharmacological treatment in vivo with the selective β_2_-agonist formoterol increased maximal mitochondrial respiration in TA. ICI pretreatment significantly blunted the increase in TA mitochondrial function induced by a single dose of ISO in mice, indicating that activation of β_2_-ARs in skeletal muscle leads to an improved mitochondrial respiration/oxygen consumption. Interestingly, only the pharmacological treatment with ISO and formoterol induced a significant increase in the ATP-independent OCR, corroborating previous data showing that sympathetic activation leads to an enhanced energy expenditure through thermogenesis and proton leak [45,46,47,48].

We have also shown that a single moderate-to-high intensity bout of AE induces significant fusion-related changes in mitochondrial morphology of TA, which was associated with increased Mfn1 protein expression and activity. It is known that mitochondrial fusion is a process that allows the exchange of enzymes, metabolites and DNA between active cells through mitochondrial membranes [49,50], and such a mechanism could be quite advantageous to skeletal muscle cells during AE, due to their high metabolic demand and the increasing probability of mitochondria damage. In fact, it has been shown that mitochondrial morphology in skeletal muscle can change in a short period of time [51], and that an acute bout of AE can be a trigger to that [52,53,54]. Although there is still no consensus in the literature on how AE modulates mitochondrial dynamics, the induction of mitochondrial fusion by AE in skeletal muscle may rely on the fact that fusion also balances mitochondrial membrane potential among organelles located in areas with different oxygen supplies, favoring ATP production in less oxygenated regions [55,56,57]. This hypothesis could explain why we did not see an AE-induced increase in mitochondrial fusion in soleus muscle (predominant oxidative metabolism), in contrast with TA, with predominant glycolytic metabolism, suggesting that mitochondrial fusion could be an efficient and faster way to improve oxidative metabolism in glycolytic skeletal muscle fibers during increased energy demand. Conversely, fragmented mitochondria (fission) are often found in resting skeletal muscle cells, with low respiratory activity, where fission contributes to maintenance of mitochondrial quality control through the elimination of irreversibly damaged mitochondria by autophagy [49,58]. Another possible explanation for these results would rely on the fact that soleus muscle has sufficient mitochondria to supply energy, whereas glycolytic muscles like TA must induce changes in metabolism to meet the high energetic demand during AE once endogenous supplies are exhausted. 

Increased mitochondrial fusion induced by AE in TA muscle was shown to be partially dependent on β_2_-AR activation, indicating that other signaling pathways triggered by exercise are collaborating to the observed morphological change. Another possibility is that a higher magnitude of sympathetic nervous activation takes place in incremental exercise versus during prolonged exercise with a sustained workload, as we used in the present study. Taking into consideration that prolonged exercise is the training method adopted for improving aerobic conditioning, our results provide evidence of β_2_-AR mediated mitochondrial fusion after a single bout of prolonged AE. To further confirm this relationship, we also observed that ISO treatment induced significant fusion-related changes in mitochondrial morphology in TA, similar to that induced by AE, which was totally prevented by ICI pretreatment, confirming that the β_2_AR subtype is the major adrenoceptor mediating mitochondrial morphological changes in skeletal muscle. Blocking β_2_-ARs during AE prevents increased Mfn1 protein expression and activity, indicating that modulation of Mfn1 function is an important mechanism by which sympathetic activity, through β_2_-AR activation, induces mitochondrial fusion in TA skeletal muscle. Indeed, TA in ISO-treated mice also showed increased Mfn1 protein expression, which was completely prevented by blocking β_2_-ARs with ICI. This response may also rely on the fact that activation of the transcription factor CREB (cAMP response element-binding) is known to increase Mfn1 protein expression through a PGC-1α-dependent manner [59,60], which is widely accepted to be induced by AE [1,61,62]. It is relevant to highlight that β_2_-ARs may contribute to induction of mitochondrial fusion while reducing fission in skeletal muscle, since DRP1 phosphorylation at Ser637 (inhibition site) by PKA inhibits its activation and its further translocation from cytosol to mitochondria [63,64]. It has been observed that high levels of cAMP can inhibit mitochondrial fission, promoting mitochondrial elongation and increasing cell viability in conditions such as fasting and increased autophagy [65]. Nevertheless, in the present study, we found no significant changes in DRP1 translocation to the mitochondria after β_2_-AR activation in TA.

β_2_-ARs couple to two different downstream signaling pathways, the canonical Gα_s_-cAMP-PKA cascade, or the non-canonical Gα_i_-arrestin cascade [42]. We tested the role of both signaling pathways in the mitochondrial response to β_2_-AR activation in vitro. First, we determined that C2C12 myotubes acutely treated with ISO increased their basal and maximal mitochondrial respiration, and that this increase in mitochondrial function was accompanied by higher ATP production by these cells, confirming our in vivo results, and demonstrating that a direct activation of β-ARs in skeletal muscle cells is also able to modulate mitochondrial function and structure in a blood flow-independent manner in vitro. Blocking β_2_-AR in myotubes significantly prevented the increased mitochondrial function and ATP production induced by ISO, showing once again that this effect is β_2_-AR-dependent. C2C12 myotubes were then acutely exposed to ISO under PKA blockade with PKI, or under Gα_i_ protein blockade with PTX, and results showed that only PKA inhibition was able to prevent the increase in mitochondrial respiration induced by ISO treatment, indicating that mitochondrial functional improvement induced during AE by β2-AR activation in skeletal muscle is dependent on the Gα_s_-cAMP-PKA classical pathway. Our findings corroborate studies that have shown the role of PKA in increasing mitochondrial function in other tissues, such as liver and heart, and in human cell lines [66,67,68,69,70]. Some evidence suggest that mitochondrial complexes, especially complexes I and IV, may be directly activated by PKA [68,71,72], which could be anchored and guided by AKAPs (A kinase anchoring proteins) from cytosol to mitochondria, where they would phosphorylate their targets [67,68,69,73]. Although investigation of the mitochondrial targets activated by PKA was beyond the scope of the present study, our results should lead to studies designed to better understand how PKA enhances mitochondrial function in skeletal muscle after acute AE, distinguishing direct and indirect interactions between PKA and mitochondria.

Taken together, we provide evidence that sympathetic activity, through β_2_-AR activation, can increase mitochondrial function and mitochondrial fusion in skeletal muscle during a single bout of AE. Altogether, we provide new insights on the molecular mechanisms whereby β_2_-AR modulates mitochondria function and structure in skeletal muscle through downstream activation of PKA and Mfn1, respectively. It is worth mentioning that these were acute responses to AE in untrained mice, and that less pronounced effects would probably be found in trained animals, as we know that sympathetic activation decreases with consistent aerobic exercise training [74,75], and that sedentary conditions are more prone to have greater responses and adaptations to exercise [76,77].

The discovery of new mechanisms associated with improved aerobic capacity induced by AE can support the development of pharmacological or gene therapy interventions that could mimic and/or maximize some of the beneficial effects of AE, for example, in patients who are unable to achieve this level of exercise. It is also worth mentioning that better elucidation of the role of β_2_-ARs in skeletal muscle could optimize the treatment of conditions such as hypertension and heart failure, and in which β-blockers are used as a therapy.

## Supplementary Materials

The following are available online at https://www.mdpi.com/2073-4409/10/1/146/s1, Figure S1: ICI treatment alone did not induce mitochondrial morphologic changes in tibialis anterior muscle.; Figure S2: Aerobic exercise did not induce mitochondrial morphologic changes in oxidative (soleus) skeletal muscle.; Figure S3: Immunoprecipitation of Mitofusin1 (Mfn1) in tibialis anterior muscle.; Figure S4: MHC Type II expression in C2C12 myotubes after 72 hours of differentiation.
