# Supplementary material for: β2-Adrenergic Signaling Modulates Mitochondrial Function and Morphology in Skeletal Muscle in Response to Aerobic Exercise
Source: Cells. 2021 Jan 13;10(1):146. doi: 10.3390/cells10010146 (PMC7828343; doi:10.3390/cells10010146)
Supplement: Supplementary file 1 [file cells-10-00146-s001.pdf]

cells

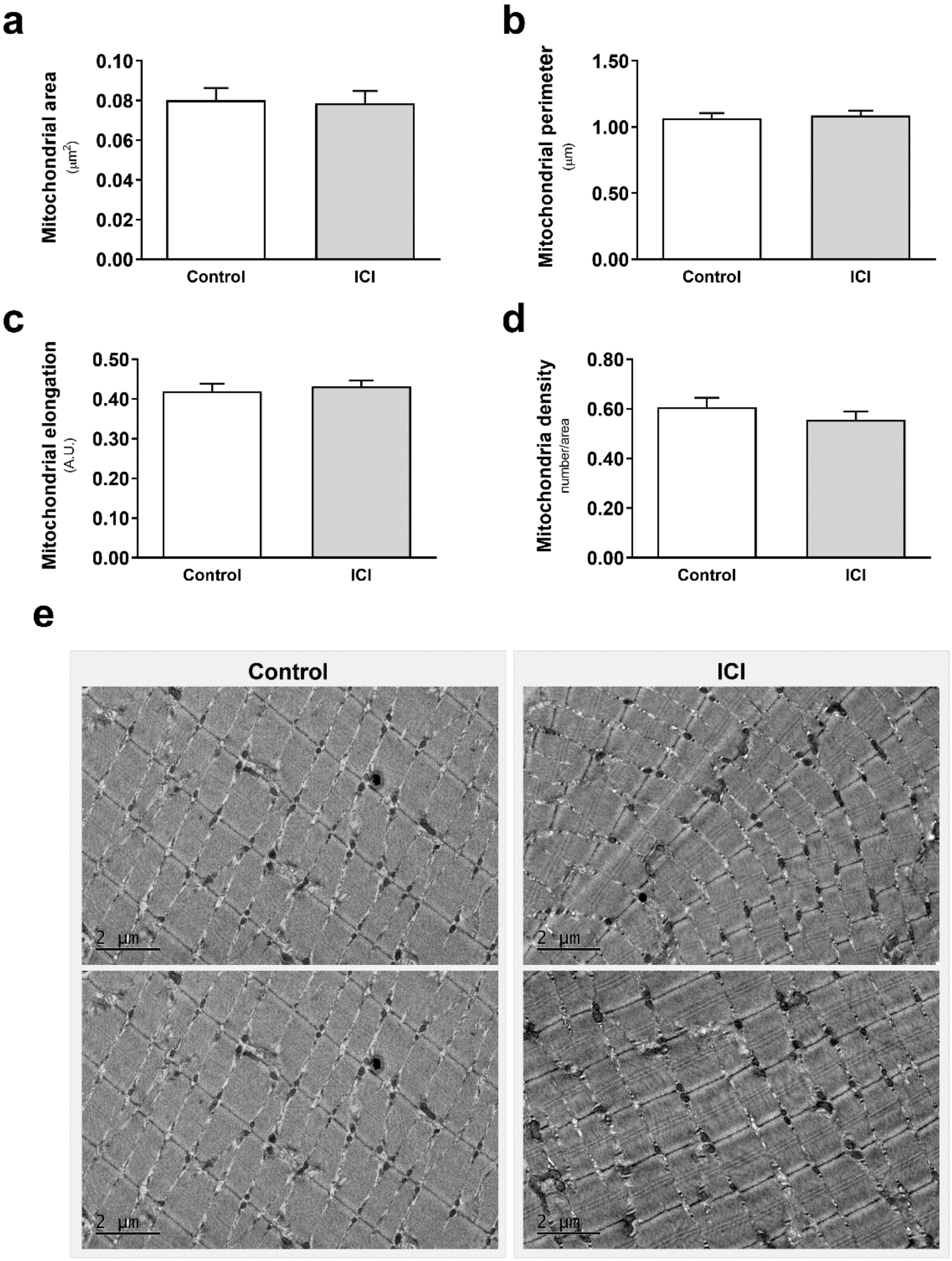

Figure 1S: ICI treatment alone did not induce mitochondrial morphologic changes in tibialis anterior muscle. Mitochondrial area (a), perimeter (b), elongation (c), mitochondria number per area (d), and electron microscopy representative images (e) of *tibialis anterior* muscle from control and ICI 118,551 treated mice (single dose - 10 mg/kg, i.p., 30 minutes prior exercise bout or ISO treatment). Data are presented as mean ± SE. n=5/group.

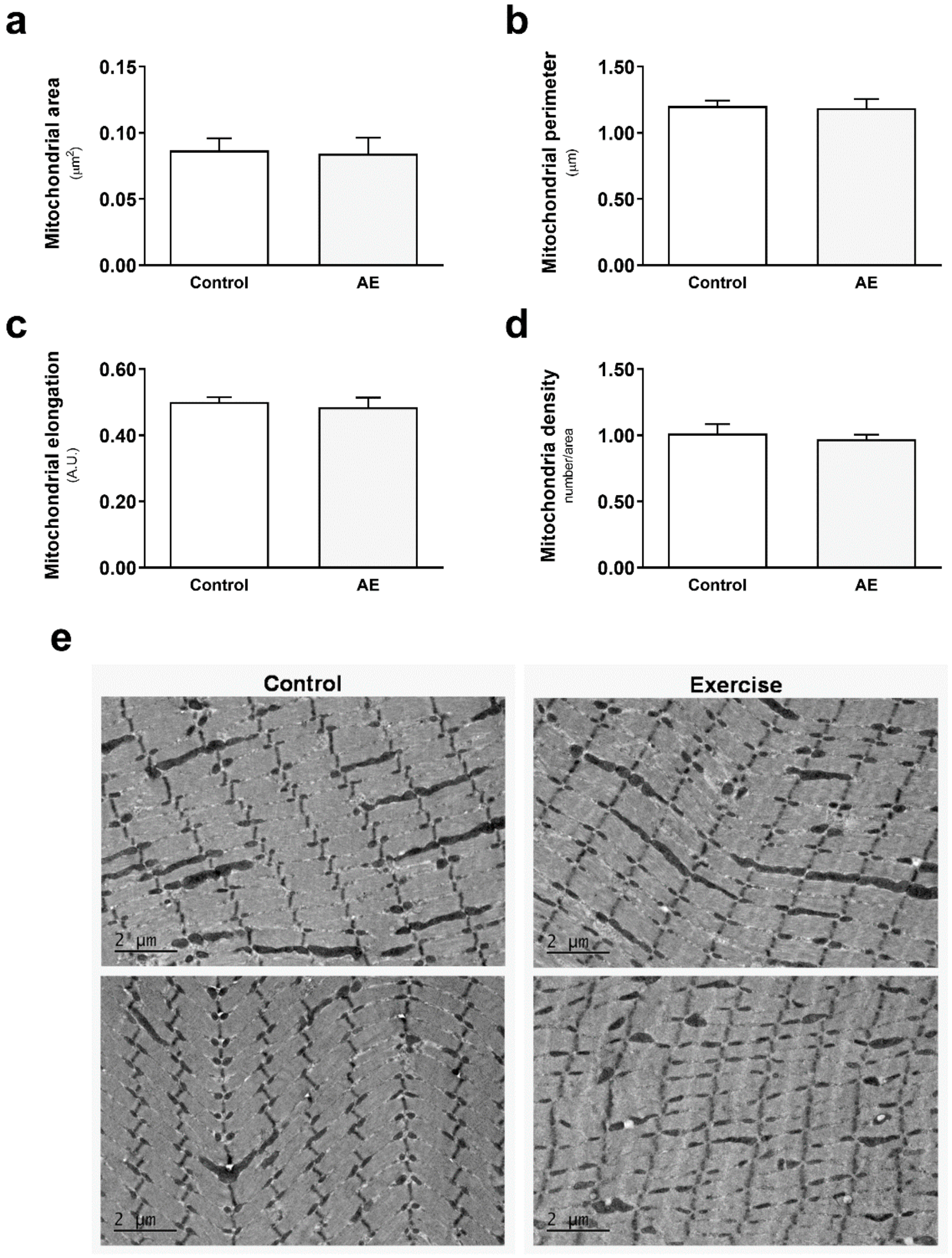

Figure 2S: Aerobic exercise did not induce mitochondrial morphologic changes in oxidative (soleus) skeletal muscle. Mitochondrial area (a), perimeter (b), elongation (c), mitochondria number per area (d), and electron microscopy representative images (e) of soleus muscle from exercised mice (single bout in a treadmill - 80% of Vmax for 1 hour, 5? inclination). Data are presented as mean ? SE. n=3/group.

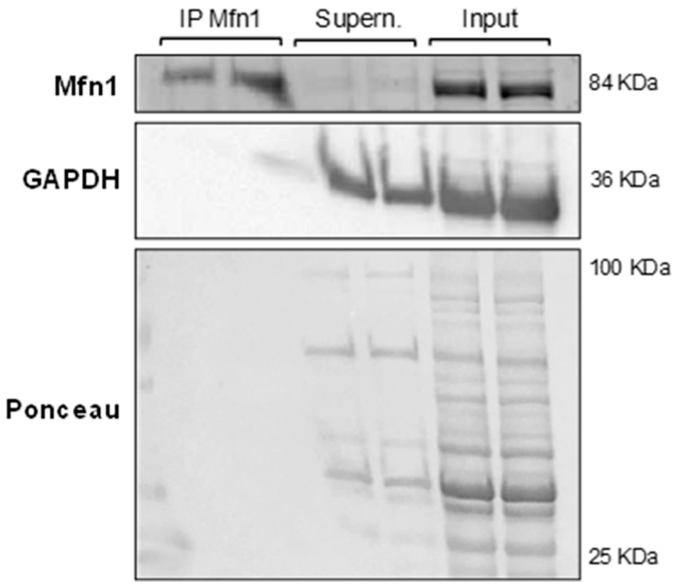

Figure 3S: Immunoprecipitation of Mitofusin1 (Mfn1) in *tibialis anterior* muscle. Representative immunoblots of Mfn1, GAPDH and Ponceau staining of purified Mfn1 by immunoprecipitation (IP Mfn1), in the remaining supernatant (Supern.) and in the total lysate (Input) of *tibialis anterior* muscle.

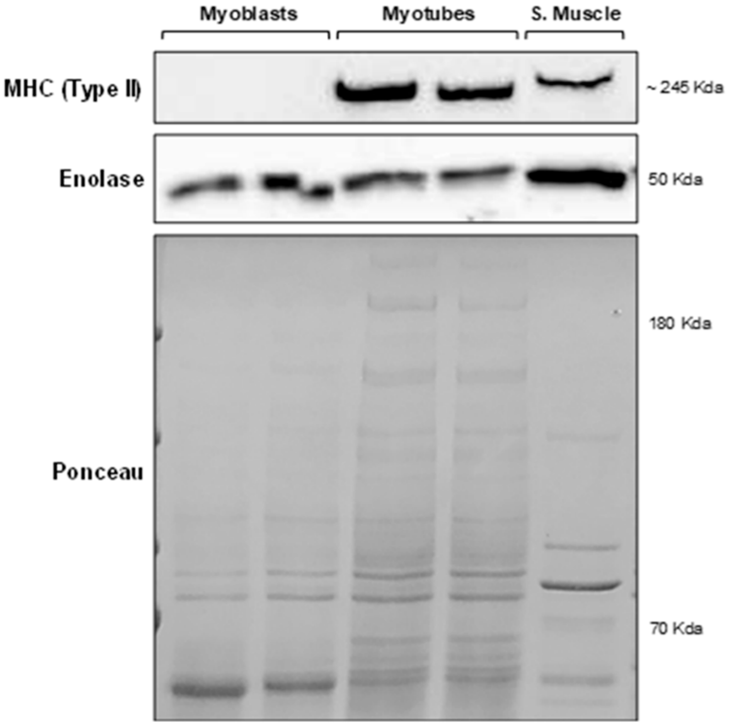

Figure 4S: MHC Type II expression in C2C12 myotubes after 72 hours of differentiation. Representative immunoblots of MHC Type II, Enolase and Ponceau staining in total lysates of C2C12 myoblasts, C2C12 myotubes after 74 hours of differentiation, and in skeletal muscle (S. Muscle).
